# Exploring phonological complexity in statistical learning of artificial words

**DOI:** 10.1371/journal.pone.0341771

**Published:** 2026-02-03

**Authors:** Akshay R. Maggu, Tobias Overath

**Affiliations:** 1 Department of Speech, Language, and Hearing Sciences, University of Connecticut, Storrs, Connecticut, United States of America; 2 Institute for Brain and Cognitive Sciences, University of Connecticut, Storrs, Connecticut, United States of America; 3 Department of Psychology and Neuroscience, Duke University, Durham, North Carolina, United States of America; 4 Duke Institute for Brain Sciences, Duke University, Durham, North Carolina, United States of America; 5 Center for Cognitive Neuroscience, Duke University, Durham, North Carolina, United States of America; Basque Center on Cognition Brain and Language, SPAIN

## Abstract

**Purpose:**

This study examined whether phonological complexity enhances auditory word learning within a statistical learning framework. Specifically, we tested if exposure to phonologically complex speech patterns (i.e., marked consonant clusters) facilitates the segmentation and generalization of both complex and simple artificial words.

**Method:**

Seventy-eight adults were randomly assigned to either a complex or simple pattern induction group and exposed to bisyllabic artificial words varying in onset complexity. Participants then heard an auditory stream containing both complex and simple words, followed by a wordlikeness rating task assessing both previously heard and novel items.

**Results:**

Exposure to complex speech patterns did not enhance wordlikeness ratings for either previously heard (stream) or novel (generalization) artificial words. Wordlikeness ratings were significantly higher for stream items than for generalization items, indicating sensitivity to exposure, but no reliable effects of induction condition or stimulus complexity were observed.

**Conclusions:**

Findings suggest that passive exposure to complex patterns does not enhance generalization within a statistical learning paradigm.

## Introduction

Speech sound acquisition is central to spoken language and remains a topic of debate across theoretical frameworks. One open question concerns the role of input complexity in driving acquisition. Complexity can be defined in various ways—phonologically (e.g., markedness), perceptually (e.g., stimulability), or statistically (e.g., transitional probabilities)—and may interact with learner-specific factors such as developmental stage or prior exposure. Some theories emphasize that acquisition proceeds from simple to complex structures based on perceptual salience, articulatory ease, or early developmental accessibility (e.g., behaviorist, connectionist, and dynamic systems theories), while others argue that exposure to complex, marked forms facilitates system-wide generalization by engaging abstract grammatical representations, e.g., [1]. A broad range of theoretical accounts address these issues, including usage-based [2], connectionist [3], dynamic systems [4], Bayesian [5], and statistical learning frameworks [6]. While these models differ in their assumptions, the present study focuses specifically on two well-defined and empirically grounded accounts—linguistic complexity, operationalized through phonological markedness, and statistical learning, defined as sensitivity to the distributional structure of speech. These frameworks offer contrasting yet complementary predictions regarding how input complexity influences learning. Our goal, in the current study, is to examine how phonological complexity may modulate the statistical learning of word-like units in an artificial language paradigm. Understanding how these two learning mechanisms jointly influence acquisition has implications for both theoretical models of language development and clinical approaches to speech sound disorders.

### Complexity in speech and language acquisition

Complexity-based theories of language acquisition, in line with generative grammar [7], natural phonology [8], and optimality theory (OT) [9], propose that exposure to complex linguistic input facilitates acquisition by restructuring the internal grammar and triggering generalization to less complex forms. Linguistic complexity in speech sound acquisition can be defined along several dimensions, including phonological markedness, lexical frequency, stimulability, and developmental consistency. Gierut [10] outlined that complexity may arise from linguistic, psycholinguistic, articulatory-phonetic, and conventional clinical factors, all of which may influence the efficacy of intervention and generalization patterns.

Phonological complexity, in particular, has been the focus of numerous speech therapy-based treatment studies [11] focused on speech production in children (3–7-year-olds) with phonological disorders (i.e., speech sound difficulties characterized by deficits in phonological representations and rule-based pattern learning, such as systematic error patterns, rather than motor articulation impairments; see [12,13]). Gierut et al. [14] conducted a seminal study comparing two approaches: training with sounds representing the child’s “most knowledge” (i.e., partially acquired) versus “least knowledge” (i.e., completely unknown). Children trained on least knowledge, or more complex, sounds demonstrated broader generalization to untreated sounds (i.e., the sounds that were not used as training stimuli), supporting the idea that complex targets result in system-wide change. Extending this framework, Gierut et al. [1] selected marked speech sounds based on implicational relationships derived from OT hierarchies. Their findings showed that speech therapy with marked sounds, such as fricatives or clusters, resulted in improvement in production of both marked and unmarked sounds, while speech therapy with unmarked sounds yielded limited generalization. Similarly, Powell et al. [15] trained children with either fricatives (more marked) or stops (less marked) and found that those trained with fricatives improved in producing both fricatives and stops, whereas those trained on stops showed limited improvement beyond the trained sounds. Further evidence comes from studies that directly manipulated the number or novelty of phonemes in treatment. Gierut [16] found that training with two new phonemes led to greater generalization than training with one new phoneme. Gierut and Neumann [17] compared homonymous and non-homonymous minimal pair therapy approaches and found that non-homonymous pairs, which were fully outside the child’s system, induced greater phonological change. Dinnsen [18] examined therapy outcomes in children trained on either marked onset clusters or less marked affricates and found that the cluster group showed more robust generalization. These findings reinforce the notion that marked or complex input engages representational mechanisms that are not triggered by simple input, leading to reorganization of the phonological system. In addition to phonological complexity, other factors such as lexical frequency and stimulability have been shown to modulate speech production outcomes. Gierut et al. [19] reported that high-frequency words, which are more complex at the sublexical level, led to greater generalization than low-frequency words. Powell et al. [20] demonstrated that treatment involving conceptual and linguistic engagement was more effective than simple motor-based articulation training. Gierut [21] showed that minimal pairs using unknown, and therefore more complex, sounds were more beneficial than those using familiar contrasts. Across these studies, complexity, whether defined by knowledge status, markedness, frequency, stimulability, or methodological strategy, has consistently been associated with greater system-wide generalization.

The effect of complexity is not limited to speech production. Maggu et al. [22] found that exposure to more marked speech contrasts in a short (15–20 minutes in total) pseudoword learning task facilitated perceptual generalization. In this study, Cantonese listeners were trained to associate Hindi prevoiced dental-retroflex contrasts (more marked) with pictures to form novel words. Those trained on prevoiced contrasts generalized to voiceless contrasts (less marked), but the reverse was not true. This finding extended the complexity effect from production to perceptual learning in typical adults. Similar complexity effects have also been observed in syntax. Thompson and Shapiro [23] demonstrated that training with syntactically complex sentence structures, such as object relatives, led to generalization to simpler structures, such as actives, while the reverse pattern did not hold. Collectively, the aforementioned findings provide strong empirical support for the complexity hypothesis. Exposure to complex structures facilitates acquisition by engaging deeper grammatical or cognitive operations and enabling generalization across untreated forms. While most of this evidence has emerged from treatment/training-based studies, the current study builds on this work by embedding linguistic complexity within a statistical learning framework to examine how these mechanisms jointly influence word learning.

On the other hand, it should be noted that not all findings support the complexity hypothesis. Rvachew and Nowak [24] found that children with phonological disorders demonstrated greater improvement when treatment targeted stimulable, early-acquired sounds compared to non-stimulable, complex ones. These results align with principles from dynamic systems theory, which emphasizes that stability in underlying components is essential for development to proceed. In the domain of speech acquisition, stimulability of simple sounds is thought to provide the foundation for acquiring more complex forms [4,25]. For example, teaching marked consonant clusters may be more successful if the child is first stimulable for unmarked plosives. Moreover, dynamic systems theory, like behaviorist accounts [26], highlights the role of reinforcement and graded exposure in shaping accurate speech patterns. Together, these findings underscore that complexity effects may be moderated by individual learner profiles and developmental readiness.

### Statistical learning in speech and language acquisition

In general, statistical learning suggests that acquisition of speech and language is a result of exposure to patterns that repeat with high probability. More specifically, it has been found that infants and children, even with minimal exposure to an artificial language, can exploit the transitional probability (TP) within a stream of sounds to find patterns that can be construed as “words” [6,27,28]. TPs are calculated as the conditional probability of one syllable following another. For example, in the four-syllable stream “prettybaby,” the TP between “pre” and “tty” (P(tty|pre)) and between “ba” and “by” (P(by|ba)) is relatively high because these syllables co-occur within real words [29]. In contrast, the TP between “tty” and “ba” (P(ba|tty)) is low, as this syllable pair straddles a word boundary. Listeners, including infants, are sensitive to these differences and are more likely to segment high-TP syllable pairs as cohesive word-like units. Indeed, infants as young as 8 months have been shown to extract trisyllabic words from continuous speech streams with only two minutes of exposure and perform above chance on segmentation tasks [30]. Further, statistical learning is a domain-general phenomenon, shown to operate not only in speech and language acquisition, but also in the domains of vision and music, and even in non-human species [30–32]. Besides the studies in typical speech and language acquisition, the statistical learning paradigm has also been used to study language acquisition in disorders such as specific language impairment [33].

Further, it has been found that statistical learning can be modulated by even prior brief exposure to phonological patterns. Along these lines, the statistical learning paradigm has been used in conjunction with pattern induction [34], i.e., it has been found that exposure to simple nonsense consonant (C) vowel (V) speech patterns facilitates parsing out the “words” from an auditory stream. Saffran and Thiessen [34] investigated how prior exposure to simple speech sound patterns facilitates SL in infants. In their experiments, 9-month-old infants were first exposed to different types of “pattern induction” stimuli—CVCV (e.g., “pada”) or CVCCVC (e.g., “padtak”) nonsense words—for 2 minutes. Then, the infants heard an artificial language stream for 1 minute containing new CVCV or CVCCVC items with statistically defined word boundaries based on transitional probabilities, following which word segmentation was evaluated using a head-turn procedure. The key finding was that infants exposed to simple CVCV patterns in the induction phase showed stronger statistical learning (i.e., better word segmentation) than those exposed to complex CVCCVC patterns. This suggested that even brief exposure to simple structural patterns can tune infants’ expectations about phonological regularities, enhancing their ability to extract words from fluent speech. Notably, learning generalized from simple to complex forms but not the reverse—posing a challenge to the complexity hypothesis, which predicts broader generalization from complex input. These results highlight the importance of input structure in scaffolding learning. However, the complexity manipulation in this study was based on syllabic structure (i.e., number of consonants), not phonological markedness. As such, while these findings show how structural simplicity can support statistical learning, they do not directly address complexity effects as defined by phonological markedness.

### Combination of linguistic complexity and statistical learning

Evidence from syntax-based studies suggests that a combination of innate rules/complexity and statistical learning might be needed to drive language acquisition [29]. Evidence from computational studies in phonology suggests that when a phonological constraint hierarchy is applied to an auditory stream, this leads to better word segmentation performance as compared to statistical learning alone [35]. However, to date, no behavioral studies have directly examined how phonological complexity, defined by markedness constraint hierarchy, interacts with statistical learning in shaping word segmentation.

To fill this gap, the current study combined statistical learning and phonological complexity (based on OT constraint hierarchy) to examine whether this combination enhanced learning and generalization beyond statistical learning alone. Specifically, we adapted the pattern induction paradigm of Saffran and Thiessen [34] and manipulated phonological complexity based on the markedness implicational hierarchy (i.e., clusters > plosives [36]). In the first stage (*Pattern Induction*), one group of participants was exposed to bisyllabic artificial words beginning with clusters (i.e., complex patterns), while the other group was exposed to bisyllabic artificial words starting with plosives (i.e., simple patterns). In the second stage (*Word Segmentation*), both groups were exposed to an auditory stream containing two new cluster-initial words (e.g.,/blɑfi/,/ʃɹubɑ/) and two new plosive-initial words (e.g.,/bɑfi/,/ʃubɑ/). In the third stage (*Wordlikeness Testing*), participants rated both previously heard words (to assess learning) and novel words (to assess generalization) on a 7-point Likert scale based on perceived wordlikeness. We hypothesized that if phonological complexity, as defined by OT constraint hierarchy (i.e., clusters > plosives) interacted with statistical learning, the participants induced with complex patterns (i.e., cluster-initial words), after listening to the auditory stream with both complex and simple words, would rate the words starting with both clusters and plosives as more word-like. On the other hand, if the phonological complexity did not interact with statistical learning, the participants induced with complex patterns (i.e., starting with clusters), after listening to the auditory stream with both complex and simple words, would rate the words starting with clusters as more word-like, but they would not rate the words starting with plosives as word-like.

## Materials and methods

### Participants

We recruited a total of 83 participants from the University of Connecticut community. They were paid at the rate of $15/hour for their participation in the study. Five participants were excluded due to being outside the normative range of hearing abilities leading to the final included sample of 78 participants (mean age: 20.47 years; SD: 3.21; 15 males, 63 females). All participants self-reported as monolingual speakers of American English, with no history of speech or language disorders, special education services, or accessibility accommodations. Participants represented a range of academic majors, including but not limited to psychology, engineering, communication disorders, biology, nursing, education, and business. The study was approved by the Institutional Review Board of the University of Connecticut (IRB # H23-0786). All participants provided written informed consent prior to participation. The recruitment start and end dates were 04/09/2024 and 27/10/2024, respectively.

A priori power analysis: A power analysis was conducted using pilot data from 10 participants exposed to complex patterns and 10 to simple patterns. The analysis determined that, to achieve a power of 0.8 with an α of 0.5 for an effect size of 0.25, a sample of 36 participants per group would be required. To account for potential attrition, the current study recruited slightly more participants than the suggested sample size.

### Hearing screening

All participants were screened for their hearing abilities for frequencies 250 Hz, 500 Hz, 1000 Hz, 2000 Hz, and 4000 Hz using a Maico MA 27 screening audiometer. Pure tones were routed via DD45 headphones. Participants with > 20 dB HL on any tested frequency were excluded from the study. Five participants did not meet this criterion and were excluded; all remaining participants met the hearing screening criterion at all tested frequencies.

### Material

Stimuli consisted of bisyllabic artificial words, either as C_m_VCV (with marked, or complex initials) or as C_u_VCV (with unmarked, or simple initials) combinations. C_m_VCV sound combinations started with clusters (e.g.,/tr/ as in “trim”) while C_u_VCV sound combinations started with plosives (e.g.,/t/ as in “tim”). Stimuli were recorded by a 25-year-old phonetically-trained male speaker using a Shure SM10A microphone and Praat [37] with a 44,100 Hz sampling rate and 16-bit resolution. Each syllable in the sound stimuli was further duration-normalized to 300 ms, intensity-normalized to 70 dB and pitch-matched to 120 Hz flat pitch contour. Table 1 lists the speech stimuli used across the three stages of the study.

**Table 1:** Stimuli (listed in International Phonetic Alphabet) used across the three stages of the study: (A) Pattern Induction; (B) Word Segmentation; and (C) Wordlikeness Testing.

(A) Stage 1: Pattern Induction								
**Complex Pattern**					**Simple Pattern**			
/blɑfi/					/bɑfi/			
/bleso/					/beso/			
/bluʃe/					/buʃe/			
/dɹɪtɑ/					/dɪtɑ/			
/dɹɑto/					/dɑto/			
/dɹɪʃɑ/					/dɪʃɑ/			
/ɡɹoʃu/					/ɡoʃu/			
/ɡlesi/					/ɡesi/			
/ɡletɑ/					/ɡetɑ/			
/plɑse/					/pɑse/			
/plofe/					/pofe/			
/plide/					/pide/			
/tɹɑɡu/					/tɑɡu/			
/tɹubo/					/tubo/			
/tɹiɡo/					/tiɡo/			
/klosɑ/					/kosɑ/			
/klofu/					/kofu/			
/kɹɑbi/					/kɑbi/			
/kɹoʃu/					/koʃu/			
/slepi/					/sepi/			
/stedu/					/sedu/			
/spoke/					/soke/			
/skɑpu/					/sɑpu/			
/ʃɹɑbu/					/ʃɑbu/			
/ʃɹupo/					/ʃupo/			
/ʃɹuki/					/ʃuki/			
/ʃɹuɡɑ/					/ʃuɡɑ/			
/fluɡi/					/fuɡi/			
/flɑko/					/fɑko/			
/flopu/					/fopu/			
**(B) Stage 2: Word Segmentation**								
**Streɑm 1**		**Stimulus**	**Streɑm 2**		**Stimulus**	**Streɑm 3**		**Stimulus**
/bɑse/		Simple	/doʃu/		Simple	/ɡifo/		Simple
/blɑse/		Complex	/dɹoʃu/		Complex	/ɡlifo/		Complex
/fudɑ/		Simple	/sebu/		Simple	/ʃubɑ/		Simple
/fludɑ/		Complex	/stebu/		Complex	/ʃɹubɑ/		Complex
**(C) Stage 3: Wordlikeness Testing**								
**Set 1**	**Type**	**Stimulus**	**Set 2**	**Type**	**Stimulus**	**Set 3**	**Type**	**Stimulus**
/doʃu/	Gen	Simple	/doʃu/	Streɑm	Simple	/ɡifo/	Streɑm	Simple
/dɹoʃu/	Gen	Complex	/dɹoʃu/	Streɑm	Complex	/ɡlifo/	Streɑm	Complex
/sebu/	Gen	Simple	/sebu/	Streɑm	Simple	/ʃubɑ/	Streɑm	Simple
/stebu/	Gen	Complex	/stebu/	Streɑm	Complex	/ʃɹubɑ/	Streɑm	Complex
/bɑse/	Streɑm	Simple	/ɡifo/	Gen	Simple	/bɑse/	Gen	Simple
/blɑse/	Streɑm	Complex	/ɡlifo/	Gen	Complex	/blɑse/	Gen	Complex
/fudɑ/	Streɑm	Simple	/ʃubɑ/	Gen	Simple	/fudɑ/	Gen	Simple
/fudɑ/	Streɑm	Complex	/ʃɹubɑ/	Gen	Complex	/fudɑ/	Gen	Complex

### Procedure

**Fig 1:**
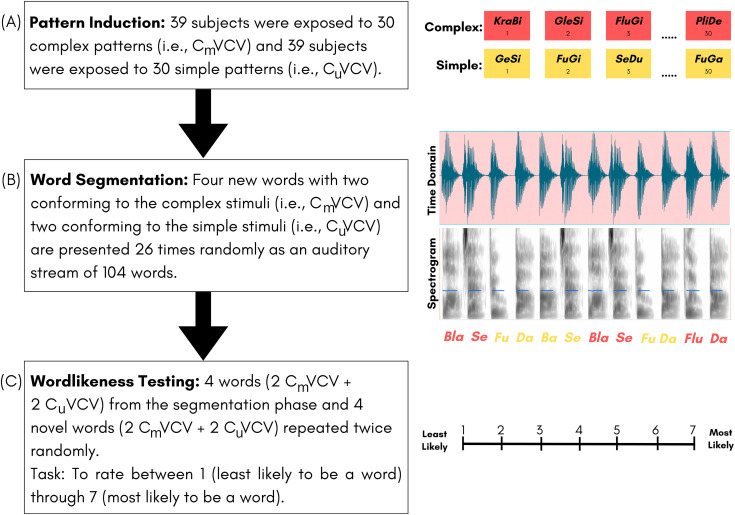
Flowchart describing the procedure of the study which entailed using the knowledge from the (A) Pattern Induction stage to (B) segment the words from the auditory stream to (C) rate the wordlikeness on a 7-point Likert scale.

The study consisted of three stages: Pattern Induction, Word Segmentation, and Wordlikeness Testing (Fig 1). The study was run using E-prime 3.0 (E-Studio Build 3.0.3.219) on a Dell laptop connected to a Roland Rubix 22 sound card and stimuli were delivered binaurally at a comfortable intensity level of 70 dB SPL via Sennheiser HD 280 Pro headphones. Participants were instructed at the start of the study to pay attention throughout the multiple stages of the study, with a special emphasis on the use of information from the first two stages for the tasks in the third stage. Fig 1 shows a flowchart of the study. The whole procedure took 5–6 minutes to complete.

#### Pattern induction.

The participants were randomly allocated to one of the two groups – Complex pattern group (n = 39; mean age: 19.94; SD: 2.4; 30 females) and Simple pattern group (n = 39; mean age: 21; SD: 3.8; 33 females). Participants in the Complex pattern group were exposed to the 30 bisyllabic speech stimuli with clusters (i.e., complex stimuli) in the initial position, while the Simple pattern group were exposed to 30 bisyllabic speech stimuli with plosives or fricatives (i.e., simple stimuli) in the initial position (Fig 1 (A)). All participants listened to a random order of presentation of the 30 stimuli. Each stimulus was 600 ms long and separated by an inter-stimulus interval of 2 s. Participants were instructed to attentively listen to the artificial words, as they would use them in subsequent stages of the experiment. However, they were not informed that the task involved identifying word boundaries. This phase was conducted without any feedback, consistent with procedures in seminal pattern induction-based statistical learning studies, e.g., [34]. Following the presentation of the 30 artificial words, the experiment moved to the second stage – Word Segmentation.

#### Word segmentation.

All participants, regardless of the patterns that they were exposed to (i.e., Complex or Simple) in the first stage, were asked to listen to an auditory stream that contained four artificial words, of which two words started with complex clusters (e.g.,/blɑfi/,/ʃɹubɑ/) and the other two started with simple plosives or fricatives (e.g.,/bɑfi/,/ʃubɑ/). These four stimuli were randomly repeated 26 times each, leading to a total of 104 stimuli presentations over a duration of 1 min and 12 secs (Fig 1 (B)). Each participant listened to one of three auditory streams with different speech stimuli (see Table 1), and within each auditory stream, one of three separate randomizations of the stimuli. Auditory streams and randomizations were counterbalanced across participants; because preliminary analyses found no differences between streams and their versions, data from the three streams were pooled for subsequent analyses. As in the Pattern Induction phase, participants were instructed only to listen attentively. As this phase was designed to promote passive exposure, no mention was made of word boundaries or statistical regularities, and no feedback was provided. Following the presentation of the 104 stimuli, the experiment moved to the third stage – Wordlikeness Testing.

#### Wordlikeness testing.

All participants, regardless of the type of patterns that they were exposed to (Complex or Simple) in the first stage, were given the Wordlikeness Testing that contained complex and simple artificial words, both old (from the Word Segmentation stage) and new (not presented in Pattern Induction and Word Segmentation stages). Specifically, stimuli in this stage consisted of 8 artificial words, with four artificial words (two simple, two complex) from the word segmentation stage (i.e., learned stimuli), and four novel artificial words (two simple, two complex) that did not appear in the word segmentation stage (i.e., generalization stimuli) (see Table 1 for details). Based on the knowledge of the stimuli that they were exposed to in the pattern induction stage and in the word segmentation stage, participants were asked to rate the stimuli in this stage on a 7-point Likert scale in terms of their wordlikeness (based on their exposure so far), with 1 being “least likely” and 7 being “most likely” a ‘word’ (Fig 1 (C)). Each word was played twice in a randomized order. The use of a rating scale rather than forced-choice recognition was intended to elicit gradient judgments of wordlikeness and reduce reliance on explicit recall or binary recognition. This measure has been used in prior work to index perceptual sensitivity to statistical structure without requiring overt segmentation judgments [38,39].

### Statistical analysis

Wordlikeness ratings were analyzed using linear mixed-effects models implemented in the *lme4* package in R. Fixed effects included Induction Group (Simple vs. Complex), Stimulus Complexity (Simple vs. Complex), Novelty (Stream vs. Generalization), and all interactions. Participant was included as a random intercept to account for repeated measurements within individuals. This modeling approach allowed all conditions to be analyzed within a single framework, rather than conducting separate analyses for stream and generalization items.

Model assumptions were assessed by visual inspection of residual distributions and residuals-versus-fitted plots. Post-hoc comparisons were conducted using estimated marginal means (*emmeans*) with Holm correction for multiple comparisons.

## Results

**Fig 2:**
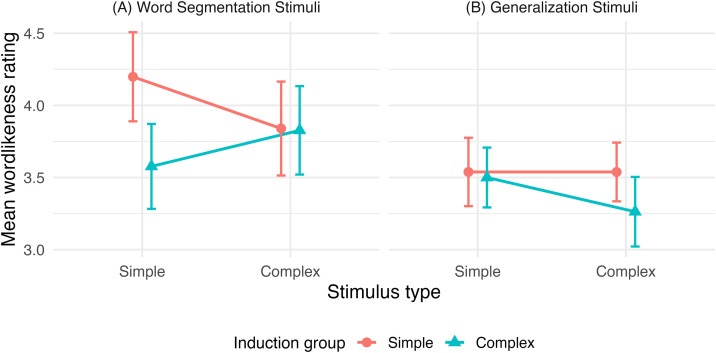
Comparison of the group exposed to Complex versus Simple patterns on the wordlikeness of the complex and simple stimuli for the (A) Word Segmentation Stimuli and for the (B) Generalization Stimuli. Error bars = ± 1 SEM.

To evaluate whether pattern induction (Simple vs. Complex) modulated wordlikeness ratings as a function of stimulus complexity (Simple vs. Complex) and novelty (Word Segmentation Stimuli vs. Generalization Stimuli), wordlikeness ratings were analyzed using a linear mixed-effects model with fixed effects of Induction Group, Stimulus Complexity, Novelty, and their interactions, and a random intercept for participant. The analysis revealed a significant main effect of Novelty, such that Word Segmentation items were rated as more wordlike than Generalization items, *F*(1, 234) = 9.42, *p* = .002 (Fig 2). No other main effects or interactions reached statistical significance (all *p*s ≥ .106). Planned contrasts indicated that induction-group differences were not significant within the Word Segmentation Stimuli condition for either simple stimuli (Simple induction: *M* = 4.20; Complex induction: *M* = 3.58; *p* = .105) or complex stimuli (Simple induction: *M* = 3.84; Complex induction: *M* = 3.83; *p* = .973), nor within the Generalization Stimuli condition (all *p*s ≥ .471). Although the Word Segmentation/Simple condition showed a numerically higher mean following simple induction, this difference did not reach statistical significance after correction.

## Discussion

In the current study, we investigated whether phonological complexity interacts with statistical learning to maximize word learning. More specifically, we examined whether exposing young adults to complex speech patterns that rank higher in the markedness hierarchy (i.e., clusters) leads to increased segmentation of both complex (i.e., C_m_VCV) and simple words (i.e., C_u_VCV) such that wordlikeness ratings would be enhanced for both complex and simple words. Overall, we found no effect of exposure of complex stimuli on wordlikeness ratings for complex and simple words, both for the words they were exposed to (i.e., stream condition) and novel words (i.e., generalization condition). Overall, wordlikeness ratings were reliably higher for items encountered in the word segmentation stage than for novel generalization items.

Contrary to predictions derived from the complexity hypothesis, exposure to complex patterns did not lead to enhanced ratings of either complex or simple stream items. Linear mixed-effects modeling revealed no significant main effects or interactions involving induction group or stimulus complexity. Although a numerical difference was observed for simple word segmentation items, this effect did not reach statistical significance after correction and did not extend to complex stimuli or generalization items. These findings suggest that, within a passive statistical learning paradigm, exposure to complex phonological structures alone is insufficient to drive system-wide generalization.

These results diverge from prior treatment-based and explicit learning studies [1,11,14,40,41] that suggest that exposure to more complex or more marked structures leads to the acquisition of both marked and unmarked structures. The main point of distinction between the current and the previous studies that showed the generalization effects of exposure to complex speech sounds on both complex and simple sounds is that the previous studies, e.g., Maggu et al. [22] used an explicit word-picture association paradigm, while the methodology of the current study involved embedding complex structures along with simple structures within a statistical learning paradigm. This difference in findings could suggest that the principles of complexity theories [42–44] are valid for explicit learning of complex structures that rank higher in the markedness hierarchy to promote acquisition of structures that are less or equivalently complex, which might not be the case when using an implicit statistical learning paradigm as in the current study.

In fact, our results are more aligned with findings from Saffran and Thiessen [34], who demonstrated that infants exposed to simpler phonotactic patterns (CVCV) were more successful in segmenting novel words than those exposed to more complex patterns (CVCCVC). In their study, learning generalized from simple to complex patterns but not the reverse, an outcome that challenges the complexity hypothesis and supports the idea that structural simplicity can scaffold early statistical learning. Similarly, in the current study, participants exposed to simple patterns exhibited numerically higher wordlikeness ratings for simple stream items and comparable ratings for complex stream items relative to participants exposed to complex patterns. Although these differences did not reach statistical significance after correction, the pattern is consistent with the idea that prior exposure to simpler structures may support the processing of more complex forms during passive exposure in an artificial language learning context.

Further, the current findings are also different from computational data on maximizing speech sound acquisition by embedding linguistic constraints within a statistical learning paradigm [35], probably due to inherent differences in the research designs of existing computational findings and the current behavioral study. The present findings can also be interpreted in the context of research on substantive bias in phonology, which suggests that learners tend to favor patterns that are more perceptually and articulatorily familiar or typologically less marked over those that are more marked [45,46]. Consequently, due to substantive bias in artificial language learning, exposure to complex or highly marked structures may not have generalized to less marked ones. Consistent with this view, the current study revealed limited evidence for complexity-driven advantages and no reliable generalization to novel items, suggesting that statistical learning in this context may be guided primarily by surface-level familiarity and distributional regularities rather than by abstract markedness relationships. This interpretation aligns with distributional learning models [47] and usage-based theories, e.g., [48], where learners extract patterns from frequent, perceptually salient input.

The current study has a few limitations: First, the participants did not have any active task to perform during the auditory stream (Word Segmentation) stage; as a result, their levels of attention could not be directly monitored. Second, no feedback was provided to the participants throughout the study, with the first two stages (i.e., Pattern Induction and Word Segmentation) not having a specific active task. Although this design was intended to promote passive statistical learning, the absence of explicit goals or reinforcement may have reduced learning strength even among attentive participants. Third, the use of wordlikeness ratings as the sole dependent measure provides only an indirect proxy for segmentation and learning, and may have been influenced by factors unrelated to statistical learning. Fourth, while the task was designed to minimize explicit awareness, we cannot fully rule out the possibility that participants developed some conscious insight into the structure of the stimuli. Finally, all stimuli were produced by a single talker; greater variability in talkers might have facilitated more robust generalization [49].

In addition, prior work using EEG and event-related potentials has demonstrated neural signatures of statistical learning that may not always be fully captured by overt behavioral measures [50–52]. These findings highlight the sensitivity of neural measures to implicit learning processes and suggest that future studies combining behavioral and electrophysiological approaches may provide a more complete characterization of how phonological complexity interacts with statistical learning.

In summary, the present results suggest that phonological complexity does not enhance generalization within a passive statistical learning paradigm. Instead, statistical learning appears to be driven primarily by exposure to encountered items, with limited transfer to novel forms. These findings refine existing accounts of complexity-based learning by delineating the conditions under which complexity effects are likely to emerge and highlight the importance of task demands and learning context in shaping phonological acquisition.
